# Rabbit models of cardiac mechano-electric and mechano-mechanical coupling

**DOI:** 10.1016/j.pbiomolbio.2016.05.003

**Published:** 2016-07

**Authors:** T. Alexander Quinn, Peter Kohl

**Affiliations:** a Department of Physiology and Biophysics, Dalhousie University, Halifax, Canada; b Institute for Experimental Cardiovascular Medicine, University Heart Centre Freiburg - Bad Krozingen, Faculty of Medicine, University of Freiburg, Freiburg, Germany; c National Heart and Lung Institute, Imperial College London, London, UK

**Keywords:** Heart, Electrophysiology, Mechanics, Sinoatrial node, Atrium, Ventricle, Slow force response, AF, atrial fibrillation, AP, action potential, BR, beating rate, Ca^2+^, calcium, Cl_swell_, swelling-activated chloride channel, ECC, excitation-contraction coupling, FSL, Frank-Starling mechanism, GsMTx-4, *Grammostola spatulata* mechanotoxin-4, H^+^, hydrogen, K^+^, potassium, K_ATP_, stretch-sensitive ATP-inactivated potassium channel, L-type, long-lasting [calcium channel], MEC, mechano-electric coupling, MMC, mechano-mechanical coupling, Na^+^, sodium, PVE, premature ventricular excitation, SAC_NS_, cation non-selective stretch-activated channel, SAN, sinoatrial node, SFR, slow force response, SR, sarcoplasmic reticulum, VF, ventricular fibrillation, V_m_, membrane potential

## Abstract

Cardiac auto-regulation involves integrated regulatory loops linking electrics and mechanics in the heart. Whereas mechanical activity is usually seen as ‘the endpoint’ of cardiac auto-regulation, it is important to appreciate that the heart would not function without feed-back from the mechanical environment to cardiac electrical (mechano-electric coupling, MEC) and mechanical (mechano-mechanical coupling, MMC) activity. MEC and MMC contribute to beat-by-beat adaption of cardiac output to physiological demand, and they are involved in various pathological settings, potentially aggravating cardiac dysfunction. Experimental and computational studies using rabbit as a model species have been integral to the development of our current understanding of MEC and MMC. In this paper we review this work, focusing on physiological and pathological implications for cardiac function.

## 1 Intra-cardiac mechano-dependent regulation

The heart is an electrically-driven pump, in which excitation of the myocardium leads to the intra-cellular release of calcium (Ca^2+^) necessary for contraction (a process commonly referred to as excitation-contraction coupling, ECC, and reviewed extensively elsewhere, *e.g*., (Bers, 2002a)). Cardiac electro-mechanical activity is extrinsically controlled in various ways, including through autonomic and hormonal inputs, but auto-regulatory mechanisms that occur within the organ itself are essential for beat-by-beat adaption to changes in physiological demand. Intrinsic control occurs *via* feed-back loops by which the mechanical state of the heart acutely alters ion channel function and/or electrical conduction (mechano-electric coupling, MEC; reviewed in (Kohl et al., 1999, Quinn et al., 2014b)), or intra-cellular Ca^2+^ handling and Ca^2+^-myofilament interactions (mechano-mechanical coupling, MMC; reviewed in (Calaghan and White, 1999, Neves et al., 2015)). By these two sets of mechanisms, the heart has distinct ways to adjust cardiac output (the product of heart rate and stroke volume) to alterations in venous return: MEC can affect heart rate (*e.g.*, through the Bainbridge response), while MMC can adjust stroke volume (*e.g.*, through the Frank-Starling and the Slow Force Responses). In addition, in cardiac pathologies associated with changes in myocardial mechanical properties and function, MEC in particular can have deleterious effects on rhythm, contributing to atrial and ventricular arrhythmogenesis (as extensively described in a comprehensive collection of works on cardiac MEC and arrhythmias (Kohl et al., 2011)).

## 2 Rabbit as a model for human cardiac electrical and mechanical function

Cardiac MEC and MMC occur at multiple levels of structural and functional integration in the heart (from subcellular to whole organ), in numerous cardiac cell types (ventricular and atrial myocytes, Purkinje and sinoatrial node (SAN) cells, and – at least for MEC – fibroblasts), and are present in vertebrates spanning the scale from zebrafish to humans. The rabbit is a particularly relevant small animal model, as its regional patterns of myocardial deformation (Jung et al., 2012), cardiac cell electrophysiology (Bers, 2002b, Nattel et al., 2008, Nerbonne, 2000), heart size to excitation wavelength ratio (Panfilov, 2006), coronary architecture (Burton et al., 2012), and response to ischaemia or pharmacological interventions (Harken et al., 1981) are much closer to human than small rodents. In as far as macroscopic electrophysiology is concerned, this advantage over other model species includes also for dog and pig (Panfilov, 2006). This has made the rabbit an important model for investigations of arrhythmogenesis and pharmacological safety testing (Hondeghem, 2016, Janse et al., 1998, Lawrence et al., 2008). In this paper we review experimental and computational studies that have used rabbit to investigate the mechanisms of MEC and MMC and their relevance for physiological and pathologically disturbed cardiac function.

## 3 Mechano-electric coupling in the heart

Studies in rabbit have been integral in forming our understanding of the relevance and mechanisms of cardiac MEC. MEC is thought to be important in both physiological and pathophysiological settings, with its role dependent on the region of the heart in which it occurs.

### 3.1 Sinoatrial node and heart rhythm

Heart excitation originates from the SAN, involving a coupled system of ion fluxes through sarcolemma (hyperpolarisation-activated ‘funny’ current, transient and long-lasting (L-type) Ca^2+^ currents, and sodium (Na^+^)/Ca^2+^ exchanger current) and sarcoplasmic reticulum (SR) membranes (DiFrancesco, 2010, Lakatta and DiFrancesco, 2009, Yaniv et al., 2015). The result is a robust system, integrating signals from multiple oscillators to allow adaptation and stability of heart rhythm in spite of changes in circulatory demand, including cyclic beat-by-beat changes in mechanical load, so that pacemaking can be viewed as a ‘clock’ driven by coupled electrical, calcium, and mechanical oscillators (Quinn and Kohl, 2012b, Quinn and Kohl, 2013a).

**Fig. 1:**
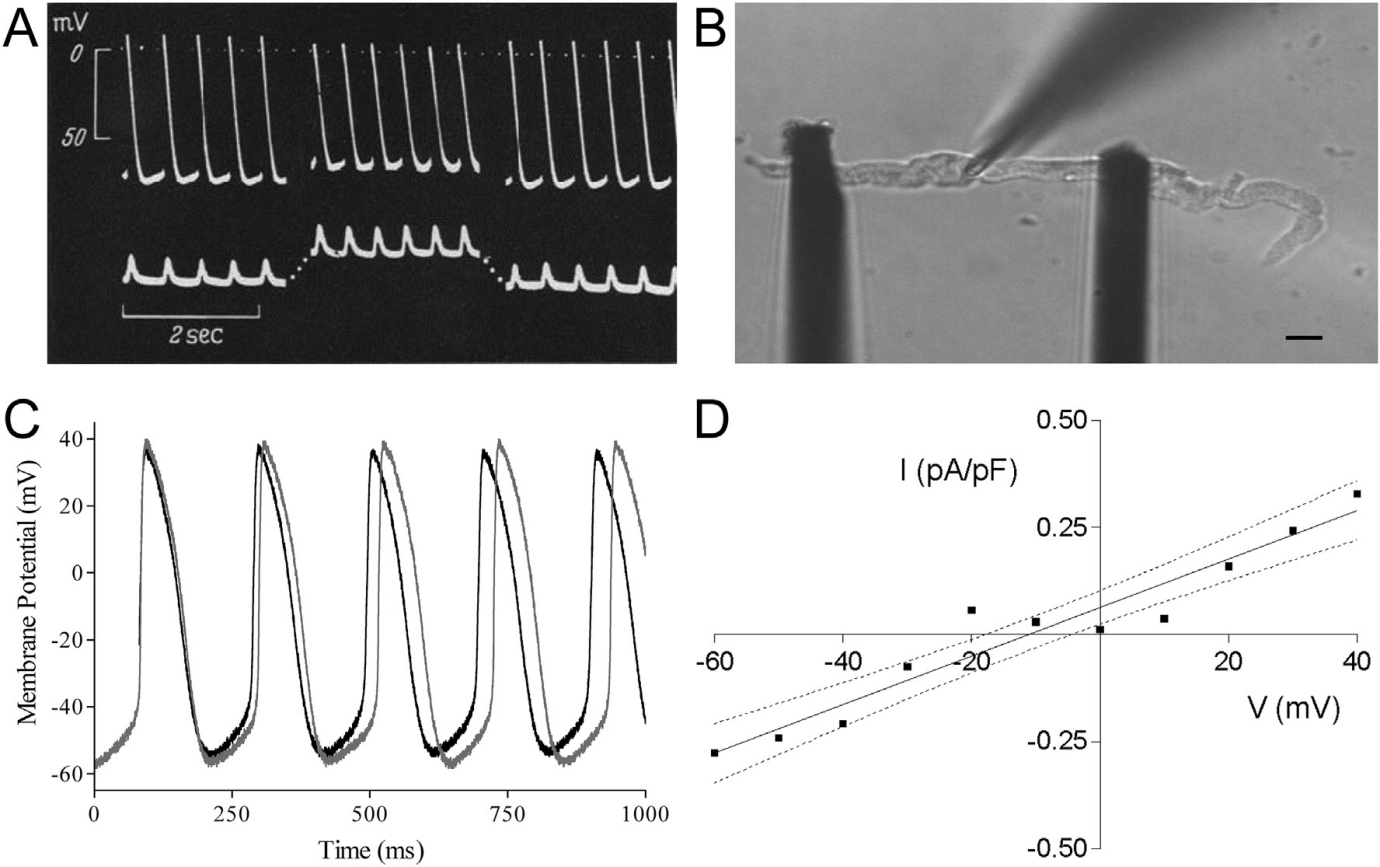
(A) Intra-cellular sharp electrode recordings of membrane potential (top) and recordings of applied and generated force (bottom) in cat isolated sinoatrial node tissue, representative of recordings taken from rabbit in the same investigation, showing an increase in beating rate with stretch (contraction pointing upwards) and reduction in absolute values of maximum systolic and diastolic potentials (from (Deck, 1964), with permission). (B) Axial stretch by the carbon fibre technique of a spontaneously beating rabbit sinoatrial node cell (right; scale bar = 10 μm) and (C) simultaneous patch-clamp recordings of membrane potential showing an increase in beating rate, accompanied by a reduction in the absolute values of maximum diastolic and maximum systolic potentials (light curve = no stretch, dark curve = stretch; from (Cooper et al., 2000), with permission). (D) Whole-cell stretch-induced current-voltage relation (difference current in absence/presence of streptomycin to block SAC) from rabbit sinoatrial node cells showing a reversal potential of −11 mV (dotted lines = 95% confidence limits, I = current, V = voltage; from (Cooper et al., 2000), with permission).

The ability of the SAN to respond rapidly to the heart’s haemodynamic status is the clearest (and, perhaps, the only well-documented) example of the physiological relevance of MEC in cardiac auto-regulation. This was recognised over one hundred years ago by Francis Arthur Bainbridge, who observed an acute increase in heart rate upon right-atrial volume-loading in anaesthetised dogs (Bainbridge, 1915), an effect known as the ‘Bainbridge Reflex’. Almost fifty years earlier, Albert von Bezold had already noted stretch-induced sinus tachycardia during increased venous return (caused by elevation of the hind legs) in rabbits with denervated hearts (Starzinsky and von Bezold, 1867), pointing to an intra-cardiac regulatory response. Fifty years after Bainbridge, John Blinks (Blinks, 1956) and Klaus Deck (Deck, 1964) showed in rabbit isolated atria and SAN (Fig. 1A) that a positive chronotropic response to stretch can be elicited in SAN tissue *ex situ*, confirming that intra-cardiac (rather than exclusively extrinsic neuronal) mechanisms are involved.

Since these seminal initial observations, results have been confirmed in rabbit isolated atria (Bolter, 1996, Cermak and Rossberg, 1988, Himmel and Rossberg, 1983, Pathak, 1958, Rossberg et al., 1985) and SAN (Arai et al., 1996, Golenhofen and Lippross, 1969, Hoffman and Cranefield, 1960, Kamiyama et al., 1984, Ushiyama and Brooks, 1977), as well as in *ex situ* preparation from various other mammalian species (Quinn and Kohl, 2012b), and it is now well established that the SAN can intrinsically respond to acute stretch on a beat-by-beat basis.

Identification of mechanisms underlying this effect has also benefitted from rabbit as an experimental model. Klaus Deck, for example, used intra-cellular sharp electrode recordings of SAN pacemaker cell membrane potential (V_m_) to demonstrate that the instantaneous increase in beating rate (BR) was accompanied by an increased rate of diastolic depolarisation and a reduction in action potential (AP) amplitude, caused by a decrease in absolute values of maximum diastolic and maximum systolic potentials (Deck, 1964). The ionic mechanisms underlying the positive chronotropic response to stretch have been investigated in rabbit isolated SAN using pharmacological agents to block swelling-activated chloride channels (Cl_swell_; using stilbene derivatives), stretch-sensitive ATP-inactivated potassium (K^+^) channels (K_ATP_; using glibenclamide), cation non-selective stretch-activated channels (SAC_NS_; using gadolinium), or to interfere with intra-cellular Ca^2+^ handling (using low extracellular Ca^2+^, block of L-type Ca^2+^ channels with nifedipine, block SR Ca^2+^ release with ryanodine, or block of Ca^2+^ re-uptake with thapsigargin) (Arai et al., 1996). That study found that the stretch-induced increase in BR can be reduced by block of Cl_swell_, by low extracellular Ca^2+^, or by inhibition of SR Ca^2+^ cycling, highlighting the interplay of sarcolemmal and SR-based pacemaker mechanisms. A similar dependence of the stretch-induced increase in BR on Ca^2+^ influx has been shown by others using verapamil as a blocker of L-type Ca^2+^ channels in rabbit atrial preparations (Himmel and Rossberg, 1983).

Initial single cell studies investigating mechanisms underlying stretch-induced changes in pacemaker rate involved positive pressure inflation of rabbit SAN cells. This has been shown to activate Cl_swell_ (Hagiwara et al., 1992) and the L-type Ca^2+^ current (Matsuda et al., 1996)). With a reversal potential near 0 mV, Cl_swell_ could theoretically account for the observed mechanically-induced changes in pacemaker electrophysiology. However, activation of Cl_swell_ usually occurs with a delay of tens of seconds after a cell volume increase, rendering it too slow for acute beat-by-beat regulation (which also, in as far as we know, is not associated with cell volume changes). Furthermore, cell inflation is mechanically different from axial stretch (cells get wider and shorter, as opposed to longer and thinner). Subsequent studies using hypo-osmotic swelling of spontaneously beating rabbit SAN cells showed that this intervention actually causes a reduction, rather than the anticipated increase, in BR (Lei and Kohl, 1998). In contrast, axial stretch (Fig. 1B) of spontaneously beating rabbit SAN cells using the carbon fibre technique (Iribe et al., 2007), results in an increase in BR (Cooper et al., 2000). This increase is accompanied by a reduction in the absolute values of maximum diastolic and maximum systolic potentials (measured by simultaneous patch-clamp recordings of V_m_ dynamics) (Fig. 1C), similar to previous reports in native SAN tissue (Deck, 1964). Subsequent V_m_-clamp studies revealed that this response was caused by a stretch-activated whole-cell current with a reversal potential near −11 mV (Cooper et al., 2000) (Fig. 1D). This current is similar to that carried by SAC_NS_ (Craelius et al., 1988, Guharay and Sachs, 1984), and could explain the observed changes in SAN BR during stretch *via* diastolic depolarisation and systolic repolarisation of SAN V_m_ (for review on cardiac SAC and their relevance for heart rhythm, see (Belus and White, 2002)). The role of SAC_NS_ has been corroborated in guinea pig and murine studies (Cooper and Kohl, 2005) demonstrating an inhibition of stretch-induced changes in SAN tissue BR by the potent SAC_NS_-specific blocker *Grammostola spatulata* mechanotoxin-4, GsMTx-4 (Suchyna et al., 2000). Of note, in murine SAN, although the ionic mechanism causing a mechanically-induced change in BR appears to be the same (SAC_NS_), a slowing of BR was seen with stretch (Cooper and Kohl, 2005). This species-difference in the response further highlights the relevance of rabbit as a model for human, in whom BR rises with an increase in venous return (Donald and Shepherd, 1978).

While rabbit isolated SAN cell and tissue experiments suggest that humoral and extra- or intra-cardiac neuronal signalling may not be pre-required for the cardiac BR response to stretch, interactions between mechanical and autonomic rate control matter. In intact rabbit (Bolter, 1994, Bolter and Wilson, 1999), as well as in rabbit isolated atria (Bolter, 1996), an increase in right atrial pressure induces both BR acceleration and a significant reduction in the percentage-response to vagal stimulation. *Vice versa*, if BR is reduced by vagal stimulation (with vagus nerve activation or pharmacological cholinergic stimulation), the chronotropic response to stretch is enhanced (Bolter, 1994, Bolter, 1996, Bolter and Wilson, 1999, Deck, 1964). It should be noted, however, that the enhanced stretch-response may also be directly related to the reduced beating rate, as when background rate is lower, stretch-induced changes in rate are increased (Coleridge and Linden, 1955, Cooper and Kohl, 2005). At the same time, the opposite effect (a decreased response to stretch) has been shown in rabbit atria with application of the muscarinic agonist β-homobetaine methylester, a structural isomer of acetylcholine (Rossberg et al., 1985). Even so, interaction of extrinsic BR regulation and intrinsic stretch-induced mechanisms may be an important mechanism for preventing excessive slowing and diastolic (over-)distension, while maintaining cardiac output and adequate circulation during haemodynamic changes that increase both venous return and arterial pressure (by invoking competing regulatory responses, *i.e.*, stretch-induced rate acceleration *vs*. the ‘depressor reflex’).

In the beating heart, stretch-induced changes in SAN function are thought to vary with timing during the cardiac cycle, being maximal in the latter part of diastole (towards the end of atrial filling), which is the very time when SAN V_m_ is moving towards the threshold for AP initiation. Stretch-induced activation of depolarising currents, such as SAC_NS_, could allow mechanical ‘priming’ of the SAN to adjust heart rate on a beat-by-beat basis in line with diastolic load. This would contribute to the matching of cardiac output to venous return (*via* beat-by-beat changes in instantaneous cycle length). Moreover, it appears that physiological loading may be essential to SAN automaticity, as slack or excessively stretched rabbit isolated SAN preparations tend to show no or irregular rhythms, respectively, while moderate preloads restore normal activity (Hoffman and Cranefield, 1960). Interestingly, stretch may also facilitate transmission of excitation from the SAN to atrium (Garny et al., 2003), as changes in BR have been shown in rabbit to correlate best with the degree of stretch in the periphery of the SAN (Kamiyama et al., 1984).

Evidence commensurate with these rabbit-derived insights into variation of heart rate with haemodynamic demand has been documented in humans. Heart rate fluctuates with the respiratory cycle, rising during inspiration (when reduced intra-thoracic pressure favours venous return) and declining during expiration (when venous return is impeded), a phenomenon known as ‘respiratory sinus arrhythmia’ (although it is a physiological response). While generally considered to be a consequence of autonomic (vagal) nervous input, respiratory sinus arrhythmia continues to exist (albeit at a reduced magnitude) in the transplanted (and thus denervated) heart (Bernardi et al., 1989). Experiments in anesthetized, vagotomised, and mechanically ventilated rabbits have confirmed that this is a consequence of sinoatrial node stretch by inspiratory increases in venous return (Perlini et al., 1995). This suggests intra-cardiac mechanisms, involving stretch-induced alterations in SAN electrophysiology that occur as a consequence of changes in venous return, are present and relevant in humans.

### 3.2 Atrial rhythm

In contrast to the apparent regulatory effects on SAN activity, MEC in working myocardium is generally thought of as contributing to cardiac arrhythmias (although physiological roles in working myocardium may exist; see ‘Future Directions’ for a brief discussion). The most common sustained arrhythmia encountered in humans is atrial fibrillation (AF) – in no small part because, in contrast to ventricular fibrillation (VF), it is not instantaneously lethal (overall incidence of new-onset AF and VF do not appear to differ (Kohl, 2013)). While many factors contribute to the initiation and progression of AF, atrial dilatation has long been causally associated with the disease, occurring in acute (*e.g.*, acute pulmonary embolus or myocardial ischaemia), transient (*e.g.*, pregnancy), and chronic (*e.g.*, mitral valve disease, hypertension and heart failure) settings (Vaziri et al., 1994).

**Fig. 2:**
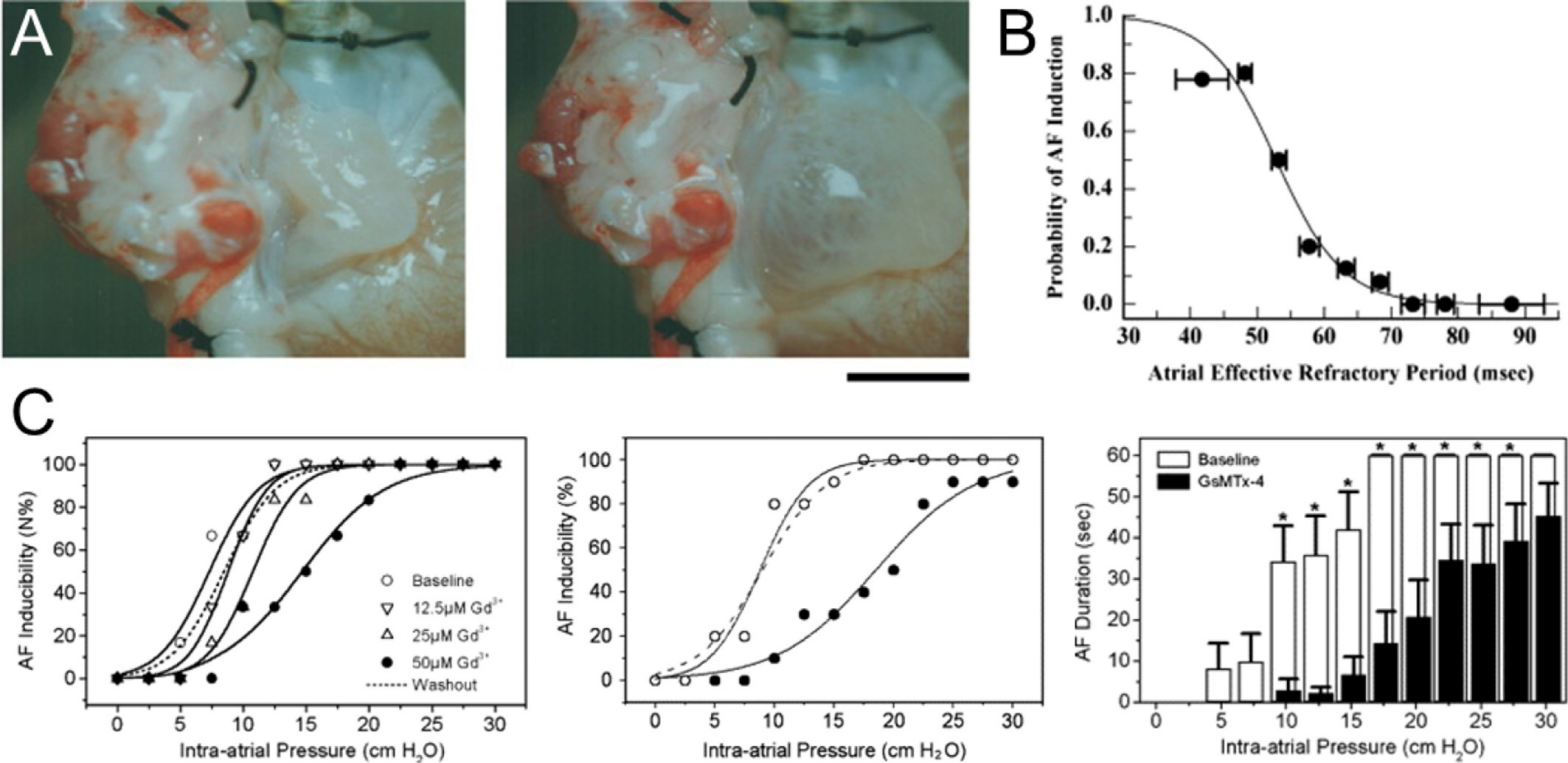
(A) Photographs of the right atrium in the isolated rabbit heart acute bi-atrial stretch model at atrial pressures of 0 (left) and 10 (right) cm H_2_O (scale bar = 1 cm; from (Ravelli, 2003), with permission). (B) Inducibility of atrial fibrillation (AF) by single premature stimulation during atrial stretch as a function of atrial refractory period (from (Ravelli, 2003), with permission). (C) Dose-dependent effect of gadolinium (Gd^3+^) on AF inducibility (left) and effect of *Grammostola spatulata* mechanotoxin-4 (GsMtx-4; 170 μM) on AF inducibility (by burst-pacing; middle) and AF duration as a function of intra-atrial pressure (* = *p* < 0.05 *vs*. baseline; from (Franz and Bode, 2003), with permission).

Atrial stretch is thought to be involved in both the initiation and maintenance of AF (Franz and Bode, 2003, Ninio and Saint, 2008, Ravelli, 2003). The isolated rabbit heart model of acute bi-atrial stretch has been instrumental in demonstrating the role of stretch in the genesis of AF. In this model, the interatrial septum of the isolated Langendorff-perfused rabbit heart is perforated, and after occlusion of the caval and pulmonary veins, biatrial pressure is increased by raising the level of an outflow cannula in the pulmonary artery (Fig. 2A). Using this preparation, it has been shown that elevated atrial pressure results in increased vulnerability to AF. This is closely related to AP shortening and a decrease in the atrial effective refractory period (Fig. 2B), which reverses within minutes of stretch release (Ravelli and Allessie, 1997). The dependence of AF inducibility on acute atrial dilatation has been recapitulated in various other studies utilising similar rabbit heart models (Bode et al., 2000, Bode et al., 2001, Chorro et al., 1998, Eijsbouts et al., 2003, Eijsbouts et al., 2004, Frommeyer et al., 2013, Li et al., 2010, Milberg et al., 2013, Ninio et al., 2005, Ninio and Saint, 2006, Ninio and Saint, 2008, Ueda et al., 2014, Xiao et al., 2010a, Xiao et al., 2010b, Zarse et al., 2001), some of which have provided additional mechanistic insight at the tissue and cellular level.

Using high-density mapping during acute right atrial dilatation by balloon inflation in isolated rabbit hearts, a global decrease of conduction velocity with stretch has been observed (Chorro et al., 1998). This slowing of conduction may be pro-arrhythmic. More importantly for the initiation and sustenance of re-entrant arrhythmias, an increase in conduction heterogeneity has also been demonstrated (Eijsbouts et al., 2003). In that study, areas of slowed conduction and lines of conduction block were identified in rabbit dilated atria, thought to relate to heterogeneous stretch of tissue with variable thickness, as is the case in particular for trabeculated regions of the atria. The resulting increase in AF inducibility can be reversed by pharmacological enhancement of gap junction conductance, while block of gap junctions causes an increase in AF inducibility by increasing total conduction time (Ueda et al., 2014). The inducibility of AF is also reduced when stretch is prevented by an intact pericardium, perhaps the most under-investigated structure of the heart (Bernardi et al., 1989), suggesting that the electrophysiological effects of acute atrial dilatation depend on tissue stretch, rather than stress (Ninio and Saint, 2006).

At the cell level, stretch will activate mechano-sensitive currents that may explain tissue-level electrophysiological changes. In fact, the inducibility of AF in rabbit heart can be reduced by altering the fatty acid composition of cardiac cell membranes by provision of dietary fish oil, possibly by changing physical membrane properties and altering mechanical stimulus transmission to mechano-sensitive currents (Ninio et al., 2005). In the stretch-augmented rapid pacing-induced AF model, both gadolinium and GsMTx-4 reduce AF inducibility in a dose-dependent manner (Fig. 2C), without affecting refractoriness (Bode et al., 2000, Bode et al., 2001, Franz and Bode, 2003), as does streptomycin (a non-specific blocker of SAC_NS_) (Ninio and Saint, 2008), suggesting a critical role of SAC_NS_. There may also be a contribution of stretch-induced excitation by SAC_NS_ from the pulmonary veins, as stretch results in an increased incidence and rate of firing, which is blocked by both gadolinium and streptomycin (Chang et al., 2007) (although with gadolinium, simultaneous block of Na^+^ channels may also contribute to suppression of excitation (Li and Baumgarten, 2001), while high concentrations of streptomycin block L-type Ca^2+^ channels (Belus and White, 2002)). The source of the decrease in refractoriness with atrial dilatation in the rabbit heart may relate to Ca^2+^ influx *via* L-type Ca^2+^ channels, as changes in refractoriness are prevented, along with the increase in AF inducibility by verapamil (although in that study, refractoriness was also reduced under conditions of minimal stretch) (Zarse et al., 2001). Alternatively, decreased refractoriness may result from K^+^ influx *via* stretch-sensitive K^+^ channels, and it has been shown that acidotic conditions (which amplify stretch activation of K^+^ channels such as TREK-1 (Maingret et al., 1999)), cause an additional reduction in refractory period and increase in AF susceptibility with atrial dilatation (Ninio and Saint, 2008).

The stretch-augmented rabbit AF model has further been used to investigate potential pharmacological therapies. Na^+^ channel block by flecainide (whose electrophysiological effects are potentiated by atrial dilatation in rabbit (Eijsbouts et al., 2004)) or ranolazine (Milberg et al., 2013) suppresses stretch-induced AF by increasing the atrial refractory period, along with inter-atrial conduction time (including in the presence of class III anti-arrhythmic agents, *i.e.*, K^+^ channel blockers such as amiodarone, dronedarone, or sotalol (Frommeyer et al., 2013)).

The importance of atrial stretch in the genesis of AF, as demonstrated in the rabbit, translates to humans, as large clinical trials established left atrial enlargement as an independent risk factor for the development of the disease (Psaty et al., 1997, Vasan et al., 1997, Vaziri et al., 1994). The fact that human data support the notion (established in rabbit models) that atrial dilatation may cause AF suggests that interventions to maintain left atrial size or target underlying mechanisms may be useful for AF prevention in the clinical setting.

### 3.3 Ventricular rhythm

As in the atria, MEC responses described in the ventricles mostly have been pro-arrhythmic, with stretch altering conduction and refractoriness, causing premature ventricular excitation (PVE) and contributing to sustained arrhythmias. This may be important in a host of cardiovascular diseases where alterations in myocardial mechanical properties may, through MEC, contribute to the electrophysiological changes responsible for arrhythmogenesis. Thus, ventricular tachyarrhythmias are frequently encountered in pathologies associated with volume or pressure overload, or changes in tissue mechanics, such as valve disease, cardiomyopathy, heart failure, hypertrophy, ischaemia, and infarction (Lab, 1982, Taggart and Sutton, 1999). Conceptually, ventricular tachyarrhythmias are thought to require a trigger and a substrate for re-entry, both of which may be generated by MEC effects (Janse and Wit, 1989, Lab, 1996, Reiter, 1996). It is difficult, however, to identify causal relationships between MEC and cardiac rhythm changes in chronic disease settings, as concurrent remodelling in tissue and cell structure and function, as well as fluctuations in metabolic and autonomic state, form confounding factors. Considering instead effects of stretch on ventricular electrophysiology in the acute setting has been an effective way to elucidate the mechanisms and potential relevance of MEC in the induction and sustenance of ventricular arrhythmias.

**Fig. 3:**
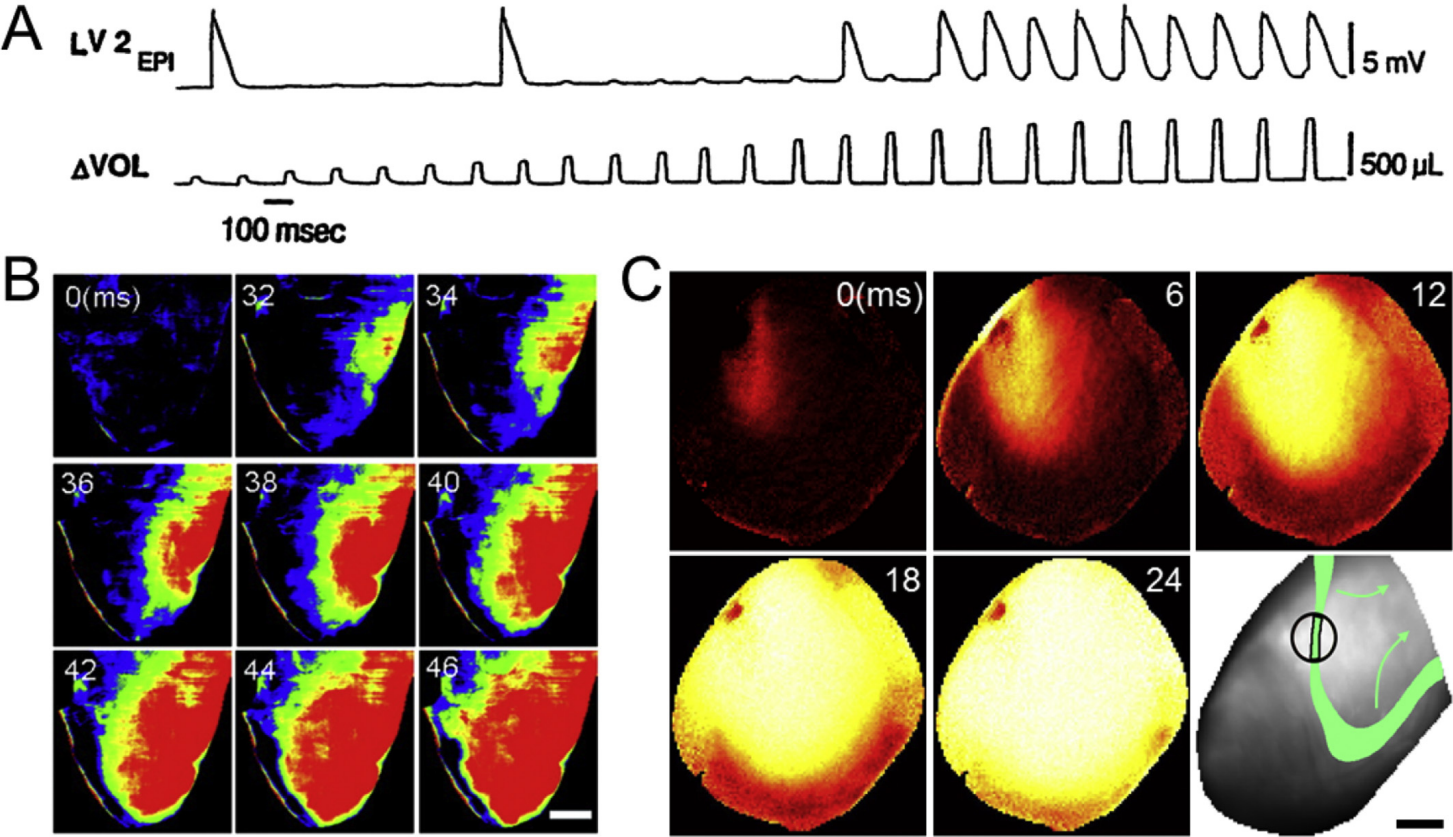
(A) Monophasic action potential recording from the left ventricular (LV) epicardium (EPI; top) during LV volume (VOL) pulses (bottom) in a rabbit isolated heart with complete heart block and rare baseline escape beats, showing transient membrane depolarisations whose amplitude increases with volume pulses, which above a certain amplitude cause ventricular excitation (from (Franz et al., 1992), with permission). (B) Voltage optical mapping of the right ventricle in a rabbit isolated heart showing focal excitation resulting from a volume pulse of 1.0 mL (scale bar = 4 mm; from (Seo et al., 2010), with permission). (C) Voltage optical mapping of the left ventricle in a rabbit isolated heart showing focal excitation resulting from sub-contusional local prodding of the epicardium, as well as the spatial interrelation of stimulation site and the 50%-repolarisation isochrone (green) in a different heart, which resulted in instantaneous VF (bottom left; scale bar = 5 mm).

A majority of experiments investigating MEC in the rabbit isolated heart have applied transient increases in intra-ventricular volume by active inflation of an intra-ventricular balloon to mechanically stimulate ventricular tissue (Bode et al., 2006, Burton and Cobbe, 1998, Dick and Lab, 1998, Eckardt et al., 2000, Franz et al., 1992, Parker et al., 2001, Parker et al., 2004, Reiter et al., 1997, Reiter et al., 1988, Seo et al., 2010, Wei et al., 2008, Zabel et al., 1996a). During diastole, whole ventricle stretch causes membrane depolarisation, which, if sufficiently large, triggers PVE (Fig. 3A) (Bode et al., 2006, Dick and Lab, 1998, Eckardt et al., 2000, Franz et al., 1992, Parker et al., 2001, Parker et al., 2004, Reiter et al., 1988, Seo et al., 2010, Wei et al., 2008, Zabel et al., 1996a). When changes in volume are applied instead during early repolarisation (a period characterised by dispersion of V_m_ in ventricular tissue), ventricular tachycardia or VF may be induced (Bode et al., 2006, Seo et al., 2010). This may be caused by a load-related decrease in the threshold for tachyarrhythmia induction (Burton and Cobbe, 1998, Reiter et al., 1988), resulting from spatially heterogeneous changes in repolarisation and refractoriness (Bode et al., 2006, Burton and Cobbe, 1998, Eckardt et al., 2000, Reiter et al., 1988, Wang et al., 2014) or conduction (Dhein et al., 2014, McNary et al., 2008). These effects are enhanced at increased BR, both in isolated hearts (Reiter et al., 1997) and in anesthetized animals (Wang et al., 2003), and continue to occur with sustained stretch (Sung et al., 2003, Zabel et al., 1996b). Interestingly, however, there appears also to be a ‘mechano-electric adaptation period’ during which subsequent intra-ventricular balloon inflations are unable to elicit PVEs (Dick and Lab, 1998), which also occurs with repetitive local epicardial mechanical stimulation (Quinn and Kohl, 2013b).

The ECG timing-dependence of mechanical stimulation, observed in rabbit and other species (Link et al., 1998), suggest that electrophysiological outcomes may depend on the spatio-temporal nature of mechanical stimulation and the underlying electrical activity. The effect of stretch timing in relation to ventricular V_m_ has been investigated in isolated rabbit heart, which demonstrated that intra-ventricular balloon inflation in diastole causes transient depolarisation, while during the AP plateau it causes repolarisation, and that these effects cross over during a transitional range of repolarisation, when stretch produces no change in V_m_ (Zabel et al., 1996a). This can be explained by SAC_NS_, as its reversal potential is about half-way between the peak and resting V_m_ of the rabbit AP (quantitatively illustrated in (Kohl et al., 1998)).

In the context of intra-ventricular balloon inflation, it is important to note that even in the setting of a global increase in volume there may be spatially heterogeneous mechanical effects, as myocardial stiffness varies throughout the ventricles with anisotropy of structure, active contraction, and passive viscoelasticity. This is apparent from isolated rabbit heart studies demonstrating that an increase in intra-ventricular volume results in non-uniform stretch, which is associated with heterogeneity of depolarisation (Seo et al., 2010). As a consequence, during increased intra-ventricular volume PVE generally originates from the area with the largest stretch, typically the ventricular free-wall and the right ventricular outflow tract (Fig. 3B) (Franz et al., 1992, Seo et al., 2010). Similarly, local stretch, applied either by inflation of an additional, localised intra-ventricular balloon (Dhein et al., 2014) or by sub-contusional epicardial impact (Quinn et al., 2011) induces PVE originating at the border of the stretched and non-stretched region (Fig. 3C) (Quinn and Kohl, 2012a). Moreover, local mechanical stimulation can result in VF when there is overlap between mechanically-induced PVE and a well-defined repolarisation wave-edge (Fig. 3C) (Quinn and Kohl, 2012a), further highlighting the importance of the spatio-temporal relation of mechanical effects and local electrophysiology.

In pathological states, this spatio-temporal dependence may be enhanced by heterogeneous changes in electrical and mechanical properties. In the ischaemic ventricle, arrhythmogenic effects of balloon inflation are increased during acute regional, but not global, ischaemia (Parker et al., 2004), again highlighting the relevance of pathophysiological heterogeneity in cardiac electro-mechanics. It has been shown that in acute regional ischaemia, physiologically-loaded, contracting rabbit hearts have a higher incidence of arrhythmogenesis than unloaded or non-contracting hearts (Lawen et al., 2015). Some ‘electrical’ diseases not thought to primarily involve mechanical dysfunction, such as long QT syndrome (which is characterised by spatially heterogeneous prolongation of repolarisation, leading to increased dispersion of AP duration, QT prolongation, and – potentially – to polymorphic ventricular tachycardia and sudden cardiac death (Roden, 2008)), may also include important contributions of MEC. Both in transgenic and pharmacological models of long QT syndrome in rabbit there is spatial correlation between regional AP duration and diastolic dysfunction (Odening et al., 2013), which provides a link between regional heterogeneity in electrophysiology and mechanics that may contribute to the associated arrhythmias (Kohl, 2013). Non-uniform mechanics may also be important for sustaining established arrhythmias, as the increase in VF frequency and complexity that is seen in the isolated rabbit heart upon stretch (Brines et al., 2012, Chorro et al., 2000, Chorro et al., 2009, Trapero et al., 2008) is enhanced by local distension (Chorro et al., 2005, Chorro et al., 2013).

Molecular mechanisms underlying the effects of ventricular stretch have also been investigated using rabbit experimental models. Stretch has been shown to cause surface membrane integration of caveolae in rabbit ventricular myocardium (Kohl et al., 2003), which may alter mechano-sensitive signalling. The role of SAC_NS_ in mechanically-induced excitation has been demonstrated by SAC_NS_ block with GsMTx-4 (Quinn et al., 2011), streptomycin (Dhein et al., 2014, Eckardt et al., 2000, Wei et al., 2008), and gadolinium (Seo et al., 2010). Streptomycin also inhibits stretch-induced changes in repolarisation and refractoriness (Eckardt et al., 2000, Wang et al., 2003) and conduction (Dhein et al., 2014) (although Na^+^ channel block with flecainide has a similar effect on conduction during stretch). The increase in the frequency and complexity of VF with stretch is similarly attenuated by block of SAC_NS_ with streptomycin (Trapero et al., 2008), as well as by block of the Na^+^/Ca^2+^ exchanger by KB-R7943 (Chorro et al., 2009), the β-blocker propranolol (Chorro et al., 2009), or the mechanical uncouplers blebbistatin and 2,3-butanedione monoxime (Brines et al., 2012). Finally, a role for the cytoskeleton in mechano-transduction has been suggested by an increase in the probability of mechanically-induced excitation by microtubule polymerization with taxol (Parker et al., 2001).

Experimental studies of MEC in the rabbit have been complemented by rabbit-specific biophysically- and anatomically-detailed computational models (Trayanova, 2011, Trayanova et al., 2010, Vetter and McCulloch, 1998), which have provided additional insight by allowing investigation of experimentally inaccessible questions, while aiding in data integration and novel, experimentally-testable hypothesis generation (Glynn et al., 2014, Gomez et al., 2015, Holzem et al., 2014, Quinn and Kohl, 2011). Studies using a three-dimensional electrophysiological model of the rabbit ventricles, including a rabbit-specific ionic model of SAC_NS_ (Healy and McCulloch, 2005), suggest that mechanically-induced changes in ventricular cardiomyocyte AP characteristics are a result of stretch in both the longitudinal and transverse myocyte axis (Vetter and McCulloch, 2001), and that changes in conduction velocity during ventricular volume loading are attributable to a reduction of intercellular resistance with a concurrent increase of effective membrane capacitance (Mills et al., 2008). A comparable model suggested, in agreement with predictions of two-dimensional simulations (Garny and Kohl, 2004), that mechanically-induced VF with sub-contusional epicardial impact may occur only when a mechanical stimulus overlaps with the trailing edge of the normal repolarisation wave (Li et al., 2004). This results in PVE-induction through SAC_NS_ in excitable tissue, next to a region of functional conduction block at the intersection of this activation and refractory tissue, and generation of potentially arrhythmia-sustaining additional heterogeneity by regional AP shortening in tissue at membrane potential levels above the reversal potential of SAC_NS_. Similarly, a three-dimensional electro-mechanical model of the rabbit ventricles has been used to illustrate the concept that in the setting of acute regional ischaemia, MEC may contribute to sustained arrhythmias by causing PVEs, conduction slowing, and unidirectional block at the ischemic border zone *via* SAC_NS_ (again, as originally predicted by two-dimensional simulations (Kohl et al., 1999)), which then allows re-entry to occur (Jie et al., 2010). These ‘wet data’-based ‘dry-model’ studies have driven subsequent experimental research in the isolated rabbit heart that confirmed several computationally-derived predictions, for example regarding the pathophysiological importance of MEC in the context of acute regional ischemia (Lawen et al., 2015) and impact-induced VF (Quinn and Kohl, 2012a).

Rabbit-specific computational modelling has also been used to investigate the implications of MEC for anti-arrhythmic therapy. Simulations have suggested that cessation of VF with precordial fist-impact may occur *via* eradication of the excitable gap by SAC_NS_-induced depolarisation (Li et al., 2006). This effect is decreased in ischaemia by stretch-augmentation of mechano-sensitive K_ATP_ activation, which reduces impact-induced depolarisation of resting myocardium and pronounces AP shortening of excited tissue, potentially even facilitating re-entry. Similarly, it has been suggested that ventricular dilatation during VF reduces the efficacy of defibrillation therapy by increasing vulnerability to electric shocks, a result of post-shock unidirectional block and re-entry through heterogeneous activation of SAC_NS_ by nonhomogeneous ventricular strain (Li et al., 2008).

These rabbit-model-derived predictions regarding the importance of ventricular volume for heart rhythm management are corroborated by observations in human. For instance, in patients with an implantable cardioverter-defibrillator, reduced ventricular volume is associated with reduced defibrillation threshold (Brooks et al., 1993, Raitt et al., 1995), such that LV dilatation is an independent predictor of a high defibrillation threshold (Gold et al., 1997), which may (at least in part) relate to a reduction in myocardial stretch (Bernstein et al., 1977). Similarly, in patients with established ventricular tachyarrhythmias, unloading of the ventricles by the *Valsalva manoeuvre* (which reduces ventricular volume as venous return to the heart is impeded during the strain phase of the manoeuvre) can temporarily restore sinus rhythm (Waxman et al., 1980). This effect can be seen even in the denervated, transplanted heart (Ambrosi et al., 1995, Taggart et al., 1992), indicating that underlying mechanisms are intrinsic to the myocardium.

On the other hand, ventricular tachyarrhythmias can be induced by increases in intra-ventricular volume, as occur during balloon valvuloplasty (Levine et al., 1988), and in heart failure there is an association between average daily median pulmonary artery pressure and arrhythmia risk (Reiter et al., 2013). Mechanically-induced tachyarrhythmias are also commonly caused by central venous and pulmonary artery catheters (Kusminsky, 2007), with incidences of up to 40% (Fiaccadori et al., 1996), by contact of intracardiac catheters and electrodes with the myocardium (Bohm et al., 2002, Lee et al., 2009, Lindsay et al., 2006), during chest compressions after electrical defibrillation (Berdowski et al., 2010), or by non-traumatic impacts to the precordium (in the setting of *Commotio cordis* (Cayla et al., 2007); a subject for which pioneering experiments over 80 years ago were performed partly in rabbits (Schlomka, 1934)). These clinical observations are analogous to observations from experiments in rabbit (discussed above) that demonstrated the induction of ventricular tachycardia or VF with transient increases in intra-ventricular volume (Bode et al., 2006, Seo et al., 2010) or by local mechanical stimulation (Quinn and Kohl, 2012a), suggesting that common MEC mechanisms may be involved.

## 4 Mechano-mechanical coupling in the heart

While rabbits have been important in investigating the sub-cellular basis of passive tension and stiffness with myocardial stretch (Bartoo et al., 1997, Linke and Fernandez, 2002), they have played a smaller role than other animal models in informing our current understanding of cardiac MMC. Still, some important insights into mechanisms leading to the rapid (Frank-Starling Law, FSL) and gradual (slow force response, SFR) increases in contractile force after stretch have come from experiments using rabbit cardiac preparations.

### 4.1 Frank-Starling mechanism

The FSL, by which stretch of myocardium results in an immediate increase in myofilament interactions and contractile force on the beat immediately following a mechanical stimulus (formalised nearly 100 years ago by Otto Frank and Ernest Henry Starling (Katz, 2002)), allows the ventricles to rapidly adjust stroke volume (and thus cardiac output) to changes in haemodynamic load. This mechanism is important not only for matching cardiac output to ‘venous return’ for the heart as a whole (as is the Bainbridge effect, acting *via* BR), but for each side of the heart individually as well. Left and right ventricular output must be matched precisely to their individual input (which can vary differently during challenges such as changes in posture or physical activity), as otherwise blood would be pooled in the pulmonary or systemic circulation. This should by no means be taken for granted, yet it works perfectly fine even in heart transplant recipients.

**Fig. 4:**
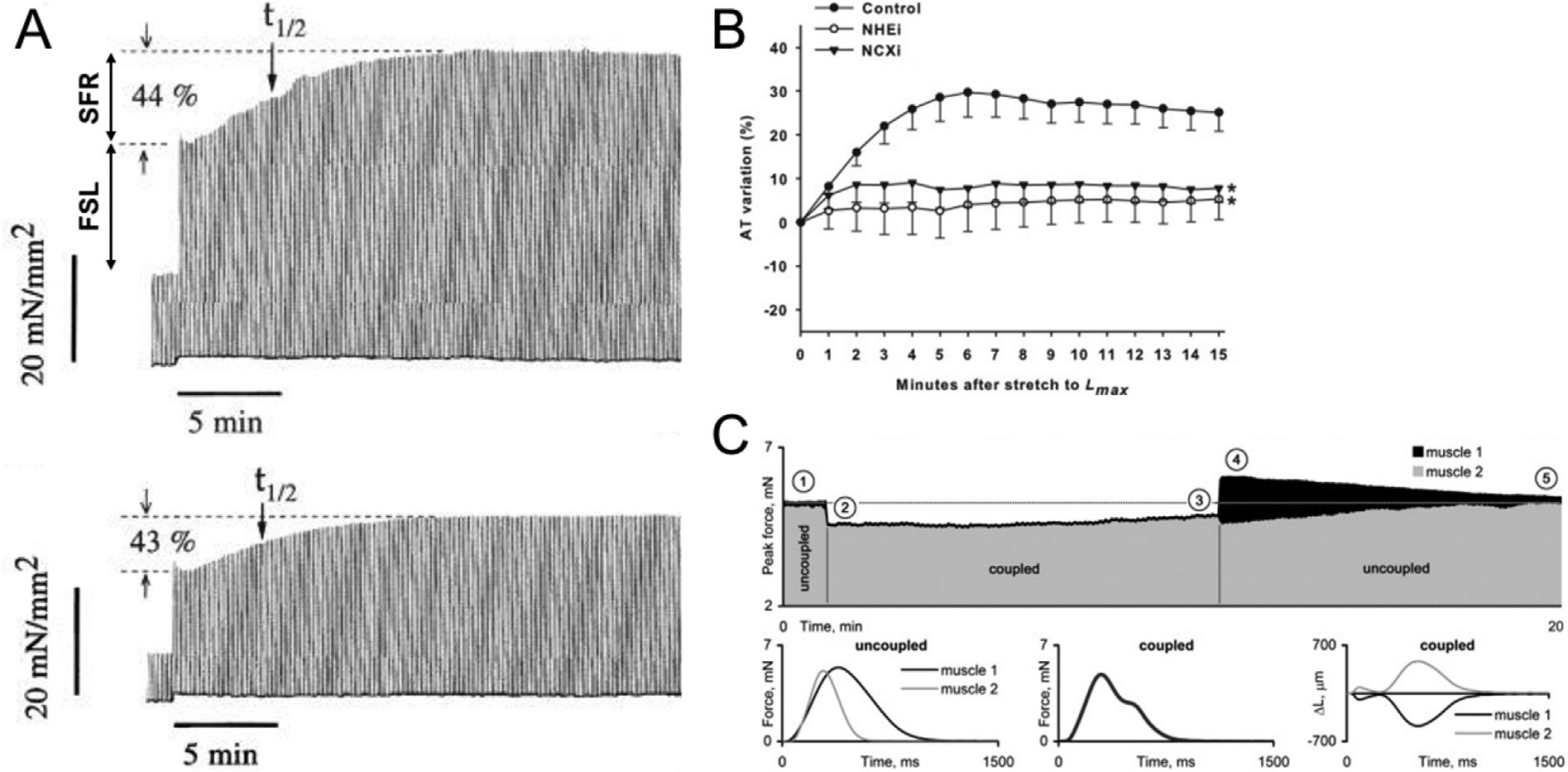
(A) Increases in rate resulting from the Frank-Starling Law (FSL) and Slow Force Response (SFR) during stretch of a rabbit isolated right ventricular papillary muscle in control (top) and with the pre-application of ryanodine (bottom; t_1/2_ = half-time of response) (from (Bluhm and Lew, 1995), with permission). (B) Modulation of the slow force response by Na^+^/H^+^ (NHEi) or Na^+^/Ca^2+^ (NCXi) exchanger inhibition (* = *p* < 0.05 *vs*. control; from (Neves et al., 2013), with permission). (C) Peak force of a pair of rabbit isolated right ventricular papillary muscles, consisting of a slow (muscle 1 at 25 °C) and a fast (muscle 2 at 30 °C) muscle with matching individual peak forces (when uncoupled). Top trace shows transitions of peak force when the muscles are uncoupled (➀), coupled in series (➁–➂, with muscle 2 stimulated with a 40 ms delay at an interval of 3 s), and after uncoupling (➃–➄, dotted line = initial peak force). Bottom traces show steady-state isometric force during a single contraction cycle before coupling (left) and when coupled in series (middle, reflected by the biphasic force development), as well as changes in length (expressed as a fraction of initial length) when coupled (right, resulting in stretch of muscle 2 by muscle 1 during contraction; from (Markhasin et al., 2012), with permission).

This immediate positive inotropic response to stretch (Fig. 4A) appears to be a result of multiple mechanisms, initially attributed to changes in thick and thin myofilament overlap, but now understood to be based on some or all of myofilament Ca^2+^-sensitivity, crossbridge- and Ca^2+^-cooperativity, inter-filament spacing, and titin-mediated strain-effects on spatial interrelations of thick and thin filaments (Ait-Mou et al., 2016, Calaghan et al., 2003, Calaghan and White, 1999, de Tombe and Ter Keurs, 2016, Neves et al., 2015). Importantly, FSL occurs immediately upon stretch, and before any increase in intracellular Ca^2+^ may be observed (although intra-cellular release of Ca^2+^ from the SR may be directly promoted by stretch (Iribe et al., 2009)). While the rabbit has not been of primary importance in delineating these effects, it was shown in rabbit isolated papillary muscle that the ascending limb of the force-tension relationship still exists when internal shortening is prevented during contraction, indicating that most of this relationship cannot be explained by variations in the degree of thick and thin filament overlap only, or by changes in the amount of filament overlap and deactivation caused by internal shortening during contraction (Julian et al., 1976). It has since been shown in the isolated rabbit heart that the Ca^2+^ sensitizer EMD-57033 increases average peak developed pressure with stretch, highlighting the possible role of myofilament Ca^2+^-sensitivity for FSL responses (Tobias et al., 1996). Intact rabbit right ventricular trabeculae have been used to demonstrate that the FSL involves effects on cross-bridge cycling kinetics, as the rate of tension redevelopment (an index of the rate of cross-bridge cycling) in intact rabbit trabeculae is decreased with stretch and depends on the predominant form of myosin (it is slower in rabbit than in rat muscle, which contains principally β- compared to α-myosin) (Milani-Nejad et al., 2013). Rabbit trabeculae have also been used to demonstrate that the βII isoform of protein kinase C is involved in phosphorylation of tropomyosin and myosin light chain-2 with stretch, such that its inhibition (by sodium trifluoroacetate) leads to a loss of the stretch-induced increase in relaxation kinetics, which alters length-dependent force generation (Monasky et al., 2013). Further, it has been shown in rabbit ventricular muscle that, in contrast to the SFR (described below), the FSL is independent of Na^+^/hydrogen (Na^+^/H^+^) and Na^+^/Ca^2+^ exchanger activity, angiotensin-II receptors 1 and 2, and protein kinase C (Neves et al., 2013), although activation of angiotensin IV receptors (by Nle1-Ang IV) in the rabbit isolated heart enhances the sensitivity of force generation to stretch (Slinker et al., 1999). For in-depth reviews on FSL and underlying mechanisms, please see (Calaghan and White, 1999, de Tombe et al., 2010, Neves et al., 2015).

### 4.2 Slow force response

The SFR refers to the gradual increase in Ca^2+^ transient amplitude and contractile force, seen over a period of minutes after the initiation of sustained stretch. Based on studies in various species, numerous ion currents and signalling molecules have been implicated in the SFR (*e.g.*, SAC_NS_, intra-cellular Ca^2+^ release, Na^+^/H^+^ exchanger, Na^+^/Ca^2+^ exchanger, Na^+^/K^+^ pump, nitric oxide, angiotensin II, endothelin, phosphatidyinositol-3 kinase, protein kinase G, and cAMP) (Cingolani et al., 2013). Rabbit studies specifically have provided essential insight into the basis for the gradual increase in Ca^2+^ transient amplitude responsible for the increase in force, as well as the role that the Na^+^/H^+^ and Na^+^/Ca^2+^ exchangers play in this effect.

While the SR is the major source of intra-cellular Ca^2+^ released on each beat, in rabbit papillary muscle inhibition of SR Ca^2+^ release (by ryanodine) (Bluhm and Lew, 1995, Kentish et al., 1992) or combined block of Ca^2+^ release and uptake (by cyclopiazonic acid) (Bluhm and Lew, 1995) has no effect on the relative magnitude of the SFR (Fig. 4A; although SFR time course is delayed by block of Ca^2+^ uptake, indicating that the SR is partly involved in removal of the extra Ca^2+^, which is in line with the increase in total SR Ca^2+^ content observed during the SFR). It has been shown in rabbit isolated ventricular muscle that the SFR is reduced by block of the Na^+^/H^+^ exchanger (by HOE 642 (Luers et al., 2005, von Lewinski et al., 2003) or 5-(*N*-methyl-*N*-isobutyl)-amiloride (Neves et al., 2013)). The SFR is almost completely abolished by reduced extracellular Na^+^ concentration (von Lewinski et al., 2003) or inhibition of the Na^+^/Ca^2+^ exchanger (by KB-R 7943; Fig. 4B) (Luers et al., 2005, Neves et al., 2013, von Lewinski et al., 2003), while it is increased by raising intra-cellular Na^+^ concentration or blocking the Na^+^/K^+^ pump (by strophanthidin) (von Lewinski et al., 2003). In contrast, block of SAC_NS_ (by gadolinium), angiotensin-II receptor 1 (by CV 11974), or endothelin-A receptors (by BQ123) were shown to have no effect in one study (von Lewinski et al., 2003), while block of angiotensin-II receptor 1 (by ZD-7155) and protein kinase C (by chelerythrine) (Neves et al., 2013) or protein kinase G (by Rp-8-Br-PET-cGMPSRp-8-Br-PET-cGMPS) (Castro-Ferreira et al., 2014) reduced the response in other reports. In addition, it has been shown that the SFR in rabbit is BR dependent (von Lewinski et al., 2008) and attenuated by ischaemia (Castro-Ferreira et al., 2014, Neves et al., 2013).

Overall, results from a range of species, including rabbit, suggest that the SFR results from increases in intra-cellular Ca^2+^ secondary to altered flux *via* the Na^+^/Ca^2+^ exchanger, in part resulting from increases in intra-cellular Na^+^ concentration mediated by the Na^+^/H^+^ exchanger (by as yet poorly defined mechanisms). There may, however, be effects acting beyond modifications in Ca^2+^ handling at the level of the myofilaments, similar to that proposed for the FSL. It has been show in ultra-thin, intact right ventricular trabeculae from rabbit that the increase in myofilament Ca^2+^ with stretch results in part from increased phosphorylation of tropomyosin, troponin I, and myosin light chain-2, on a time scale that would implicate it in the SFR (rather than in the FSL) (Monasky et al., 2010).

As well as a means for further increases in force generation above the FSL, the SFR may represent an important auto-regulatory mechanism for normalising force generation in mechanically heterogeneous myocardium (similar to the inter-cellular matching of contractile force by a stretch-induced increase in Ca^2+^ spark rate (Cannell, 2009)). Experiments using duplexes of dyssynchronously interacting rabbit ventricular papillary muscles have demonstrated slow and opposite changes in the peak force and in shape and duration of Ca^2+^ transients when one underwent shortening and the other lengthening during mechanically-coupled contractions (Fig. 4C; (Markhasin et al., 2012)). This was associated with changes in calcium load, and may explain the matching of local contractility of ventricular myocytes to global mechanical demand.

In human, one of the most common challenges to cardiac function is an acute increase in haemodynamic load. This occurs in physiological settings (*e.g.*, changes in posture or exercise) and with numerous pathologies (*e.g.*, hypertensive crisis, valve prolapse, or acute heart failure), and requires the heart to respond with a rapid increase in cardiac output. For instance, the enhanced venous return that occurs with exercise (a consequence of increased skeletal muscle and respiratory activity) leads to increased volume of the cardiac chambers (Nobrega et al., 1995). Without the mechanical response to stretch, as described in rabbit (and various other species, including human), this would have negative energetic implications, as according to the Law of Laplace, more force would need to be generated by individual cells in a larger chamber to achieve the same pressure levels. If not countered by FSR and, if that is not sufficient – SFR, changes in preload could lead to pulmonary and/or systemic congestion. Thus, the FSL and SFR (changing stroke volume) and Bainbridge response (changing BR), are key regulators of normal cardiac output, and active in the denervated (transplanted) heart.

## 5 Future directions

Studies using experimental and computational models of rabbit heart have been drivers of progress in our current understanding of the physiological importance and mechanisms of mechano-electric and mechano-mechanical interactions in the heart. Clinically-relevant MEC and MMC mechanisms and responses are beginning to be identified (see collection of works by leading investigators in (Kohl et al., 2011)), as well as a physiological role for these effects (Cannell, 2009, Iribe et al., 2009, Opthof et al., 2015, Quinn, 2015, Rhodes et al., 2015, Solovyova et al., 2014), with potential for translation to novel mechanics-based therapies. Interestingly, MEC effects on working myocardium have thus far been mainly considered in the context of electrical disturbances. This is likely to be a side-effect of the actual physiological relevance of MEC mechanisms, which – *via* changes in trans-sarcolemmal Ca^2+^ flux balance (acting directly on Ca^2+^ or indirectly *via* Na^+^ and potentially facilitated by a mechanically-mediated change in transverse tubule-extracellular exchange (McNary et al., 2012)) – may be a key mechanism that allows individual cells in the myocardium to adjust their contractility to systemic demand. After all, every single cardiomyocyte is activated on every beat of the heart, and any change in hemodynamic demand will have differential and non-linear effects on single cell force-length dynamics (Wang et al., 2011). This is perhaps where MEC and MMC meet, and further research into the interrelation of mechano-sensitive changes in electrical and mechanical activity is clearly needed.

In this process, we expect to see improvements in the delineation of therapeutically-targetable subcellular structures and of mechanisms underlying pathological effects, such as mechano-transduction pathways (Kerr et al., 2015, Traister et al., 2014) and the ion fluxes involved (Belus and White, 2002, Reed et al., 2014). Similarly, the pathological importance of tissue-level effects, such as cardiac myocyte-non-myocyte biophysical interactions (Gourdie et al., 2016, Kohl and Gourdie, 2014, Quinn et al., 2014a) or changes in cardiovascular mechanics with disease (Quinn, 2014, Quintanilla et al., 2015), are key research targets. These need to be linked to extra-myocardial biophysically-relevant structures, from heart valves to pericardium, and from intracardiac blood-flow dynamics to interactions with the vasculature (Hunter et al., 2003, Nordsletten et al., 2011). In this process, rabbit-based ‘wet’ and ‘dry’ model systems with their high relevance for human cardiovascular structure and function, including the growing potential for genetic modification (Duranthon et al., 2012, Peng, 2012)), will undoubtedly be a major player.

## Editors’ note

Please see also related communications in this issue by Kang et al. (2016) and Rog-Zielinsky et al. (2016).
